# MiR-33a functions as a tumor suppressor in triple-negative breast cancer by targeting EZH2

**DOI:** 10.1186/s12935-020-1160-z

**Published:** 2020-03-18

**Authors:** Zeng Weihua, Zou Guorong, Cao Xiaolong, Li Weizhan

**Affiliations:** Department of Oncology, Panyu District Cancer Institute, Guangzhou Panyu Central Hospital, No. 8, Fuyu East Road, Qiaonan Street, Panyu District, Guangzhou, 511486 People’s Republic of China

**Keywords:** TNBC, miR-33a, EZH2, Proliferation, Mobility

## Abstract

**Background:**

Increasing reports have confirmed that microRNAs play an important role in breast cancer progression, particularly in triple-negative breast cancer (TNBC). The aim of our study was to investigate the role of miR-33a in TNBC progression.

**Methods:**

PCR assays were performed to detect miR-33a and EZH2 expression in TNBC tissues, adjacent nontumor tissues and cell lines. Western blot, CCK8, Transwell, cell colony formation and EdU cell proliferation, cell cycle analysis and luciferase reporter assays were used to determine the regulation of miR-33a/EZH2 in TNBC progression.

**Results:**

MiR-33a was significantly downregulated in TNBC tissues and cell lines. MiR-33a overexpression in TNBC cells significantly inhibited cell growth and mobility and induced G1 cell cycle arrest. The luciferase reporter assay revealed that EZH2 is a direct target of miR-33a and that it was upregulated in TNBC tissues and cell lines. There was a negative correlation between miR-33a and EZH2 expression in TNBC tissues. EZH2 knockdown exerted similar inhibitory effects, while ectopic expression of EZH2 showed suppressive effects on malignant behaviors induced by miR-33a overexpression in TNBC cells.

**Conclusions:**

These findings revealed that miR-33a is a tumor-suppressive miRNA in TNBC and can inhibit proliferation and mobility and induce G1 cell cycle arrest by directly targeting EZH2.

## Background

Breast cancer (BC) is the most frequent and highly lethal tumor in women, and triple-negative breast cancer (TNBC) accounts for approximately 10–24% of BCs [1, 2]. TNBC, the most aggressive type of BC with a high proliferative and metastatic phenotype, is characterized by the absence of expression of the estrogen receptor (ER), progesterone receptor (PR) and human epidermal growth factor receptor 2 (HER-2) [1, 3]. TNBC has a worse prognosis, higher histological grade and displays high rates of drug resistance, metastasis and prost-surgical reoccurrence [4–7]. To date, there are still no efficient agents for the treatment of TNBC because of a lack of specific therapeutic targets [8, 9]. Therefore, it is urgent to identify novel specific regulators in TNBC, which might provide new insights and clues for the development of more effective therapies for TNBC.

MicroRNAs (miRNAs) are a class of highly conserved small noncoding single-stranded RNA molecules [10]. They can regulate a variety of cellular processes, including cell proliferation, mobility, differentiation and metabolism, by base-pairing with the 3′-untranslated regions (3′-UTRs) of target genes [11]. An increasing number of studies have revealed that miRNA dysfunction contributes to BC development and metastasis [12, 13]. MiR-33a downregulation has been associated with cancer cell proliferation, mobility and chemotherapy sensitivity in various cancers, including lung cancer [14, 15], prostate cancer [16] and BC [17, 18], which suggested that it functions as a tumor suppressor. MiR-33a was upregulated after treatment with chidamide in TNBC and suppressed glycolysis by targeting LDHA [19]. It can also target ADAM9 and ROS1 to suppress BC proliferation and metastasis [18]. However, the detailed mechanisms of miR-33a in TNBC proliferation and mobility remain unclear.

The polycomb group protein enhancer of zeste homolog 2 (EZH2) is a histone-lysine *N*-methyltransferase enzyme that regulates DNA methylation and suppresses RNA transcription [20, 21]. EZH2 is highly expressed in many kinds of cancers including BC [22], and EZH2 can regulate TNBC tumor growth and metastasis [23–25]. Knockdown of EZH2 suppressed TNBC MDA-MB-231 tumor growth and metastasis in xenograft models [26, 27]. Tumor-suppressive miRNAs that directly target EZH2 to inhibit TNBC progression include miR-1301 [28] and miR-340 [25]. It has also been reported that EZH2 expression in TNBC patients is correlated with aggressiveness, advanced tumor stage and increased mortality [29, 30]. Therefore. EZH2 inhibition might be a promising potential therapeutic target for the treatment of TNBC. Whether EZH2 expression is associated with miR-33a levels in TNBC has not been determined.

In our study, we analyzed the expression of miR-33a and EZH2 in TNBC tissues and determined the role of miR-33a and EZH2 in TNBC progression. Our resulted uncovered an important role of EZH2 in the growth and mobility of TNBC cells and confirmed EZH2 was a target of miR-33a. Thus, targeting miR-33a/EZH2 signaling may be a potential strategy for TNBC treatment.

## Materials and methods

### Clinical specimens

TNBC tissues (n = 60) and adjacent nontumor tissues (n = 30), which included 15 TNBC tissues and 15 matched adjacent nontumor tissues from the same patients, were surgically obtained from Guangzhou Panyu Central Hospital. The tissues were stored in liquid nitrogen after collection, and all specimens were confirmed by pathological examination. Prior patient consent and approval from Guangzhou Panyu Central Hospital were obtained for the use of these clinical tissues in the study. This research was authorized by the Ethics Committees of Guangzhou Panyu Central Hospital (ethical number: NN-WK-2016115).

### Cells and cell culture

The human triple-negative breast cancer cell lines MDA-MB-231, MDA-MB-453, MDA-MB-468 and BT-549 and human normal breast epithelial cell line MCF10A were purchased from the Cell Bank of Type Culture Collection of Chinese Academy of Sciences (Shanghai, P.R. China). All the cells were cultured in recommendatory culture media containing 10% FBS and 1% penicillin-streptomycin and maintained in a humidified atmosphere of 5% CO_2_ at 37 °C.

### Cell transfection

For gene knockdown experiments, MDA-MB-231 and BT-594 cells were transfected with EZH2 siRNA (GenePharma) by Lipofectamine™ RNAiMAX Transfection Reagent (Invitrogen) according to the protocol. For gene overexpression experiments, the cells were transfected with pCMV3-*EZH2* plasmid, or miR-33a mimic by Lipofectamine™ 3000 Transfection Reagent (Invitrogen). After a 6-h transfection, medium containing transfection reagents were refreshed and cells were further cultured with fresh medium for 24 h. The knockdown of EZH2 was performed with following siRNA duplex: 5′-GAGGGAAAGUGUAUGAUAATT-3′ and 5′-UUAUCAUACACUUUCCCUCTT-3′.

### Quantitative real-time PCR

Total RNA was extracted from TNBC tissues and cell lines using the E.Z.N.A.^®^ Total RNA Kit I (Omega Bio-Tek) according to the manufacturer’s protocol. Reverse transcription was performed with the Transcriptor First Strand cDNA Synthesis Kit (Roche). Then, cDNA was amplified and quantified with the LightCycler 480 Real-Time PCR System (Roche) using 2× SYBR Green I Master Mix (Bimake). For miRNA quantification, total RNA was reverse transcribed with the PrimeSript miRNA cDNA Synthesis Kit (TaKaRa), and the miR-33a cDNA was amplified and quantified with the LightCycler 480 Real-Rime PCR System (Roche) using 2 × SYBR Green I Master Mix (Bimake). The levels of mRNA and miRNA were normalized to *ACTB* and U6 levels, respectively. The 2^−ΔΔCT^ method was used to determine relative gene expression.

### Western blot assay

Total cell protein of TNBC cells was extracted by cell lysis in RIPA buffer (Thermo Fisher Scientific) containing protease and phosphatase inhibitor cocktails (Bimake). The protein concentrations were detected by a BCA Protein Assay Kit (Invitrogen). Proteins were separated by SDS-PAGE, transferred onto polyvinylidene difluoride (PVDF) membranes (Millipore) and subjected to immunoblot analyses. The blot was performed with primary antibodies against EZH2 and GAPDH (1:1000 dilution. Cell Signaling Technology). The signals were detected by an HRP-conjugated secondary antibody (1:2000 dilution. Cell Signaling Technology) and the bands were visualized with an enhanced chemiluminescence (ECL, Millipore) system according to the manufacturer’s protocol.

### Cell proliferation assay

The effects of miR-33a and EZH2 on the proliferation of MDA-MB-231 and BT-549 cells were measured by the EdU cell proliferation assay (Beyotime Biotechnology) and CCK-8 assay (Beyotime Biotechnology). Briefly, cells were seeded in 96-well plates and cultured overnight. Cells were transfected with miRNA mimic, siRNA or plasmids for 6 h, and the medium was replaced. For the EdU cell proliferation assay, cells were subjected to an EdU cell proliferation assay kit according to the standard protocol at 24 h. The images were acquired with an inverted fluorescence microscope. For the CCK-8 assay, the absorbance at 450 nm was measured using a microplate reader at 0, 24, 48, 72, and 96 h.

### Colony formation assay

Colony formation can be used to evaluate cell proliferation capacity. MDA-MB-231 and BT-549 cells were transfected with miRNA mimic, siRNA or plasmids for 24 h, and then the cells were trypsinized and seeded into 6-well plates at approximately 1000 cells per well. After culture for 10 days, the cells were fixed with 4% paraformaldehyde and stained with 0.1% crystal violet. The visible colonies of cells were observed and counted.

### Cell cycle analysis

The effects of miR-33a and EZH2 on the cell cycle in MDA-MB-231 and BT-549 cells were detected using flow cytometry analysis. Cells were transfected with miRNA mimic, siRNA or plasmids for 48 h, trypsinized, fixed with pre-cooled 70% ethanol and treated with 1 mg/mL RNase for 30 min at 37 °C. Then, the intracellular DNA was labeled with propidium iodide (PI) (Beyotime Biotechnology) for 30 min at 4 °C and analyzed by a flow cytometer (BD). The populations of TNBC cells in G1, S and G2/M phases were calculated with ModFit software (Verity Software House Inc., Topsham, ME, USA).

### Luciferase reporter assay

The 3′-UTR of EZH2 containing miR-33a binding sites and its mutant were cloned into the pGL3-control luciferase reporter vector. The pGL3-EZH2 or mutant pGL3-EZH2 plasmid was co-transfected with miR-33a or NC mimics into MDA-MB-231 and BT-549 cells. After a 48-h transfection, luciferase activity was evaluated by the Dual-Luciferase Reporter Assay System (Promega) and was normalized to the activity of *Renilla* luciferase driven by a constitutively expressed promoter in the phRL vector.

### Cell migration and invasion assay

The effects of miR-33a and EZH2 on the migration and invasion of MDA-MB-231 and BT-549 cells were measured by Transwell assays (Corning). For the Transwell migration assay, TNBC cells were first transfected with miRNA mimic, siRNA or plasmid. After transfection for 24 h, cells were trypsinized, resuspended in serum-free medium and added into the upper filters. The lower chambers were filled with complete culture medium. After a 24-h incubation, the migrated cells were fixed with 4% paraformaldehyde and stained with 0.1% crystal violet. The cells on the inner sides of the upper filters were removed with cotton swabs, and the migrated cells on the bottom sides were imaged. For the invasion assay, the upper filters were precoated with diluted Matrigel (Corning), and the following procedures were the same as those for the Transwell migration assay.

### Statistical analysis

Each experiment was repeated three times, and all statistical analyses were performed using GraphPad Prism 8.0 software. Data are presented as the mean ± SEM. The differences between two groups were determined by two-tailed Student’s *t* test, and differences among more than two groups were analyzed using one-way ANOVA followed by the Tukey test. *P* < 0.05 indicates a significant difference.

## Results

### miR-33a is downregulated in TNBC tissues

**Fig. 1:**
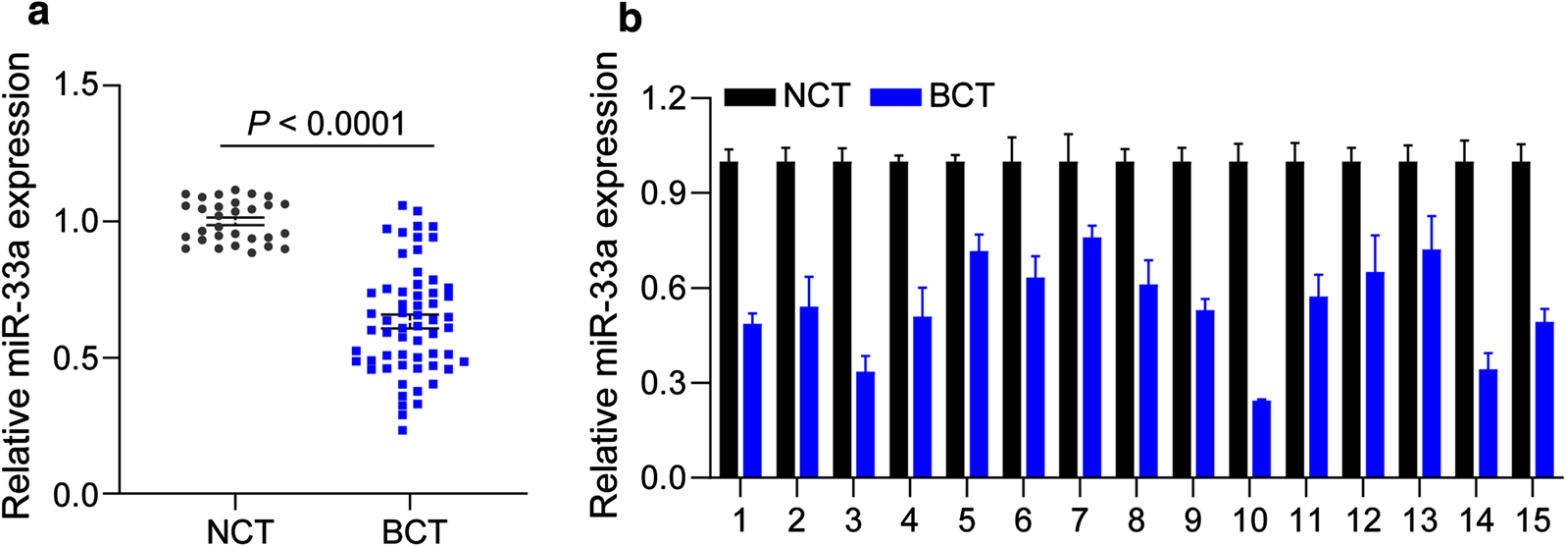
miR-33a is downregulated in TNBC tissues. **a** The expression of miR-33a in 15 TNBC tissues and matched adjacent nontumor tissues was detected by PCR assay. **b** miR-33a expression was significantly lower in TNBC tissues (n = 75) than in adjacent nontumor tissues (n = 30). Data are shown as mean ± SEM

We first investigated the expression of miR-33a in TNBC tissues and adjacent nontumor tissues by qRT-PCR assay. As shown in Fig. 1a, the expression of miR-33a was significantly downregulated in TNBC tissues, compared to adjacent non-tumor tissues (*P* < 0.001). We also detected miR-33a expression in 15 pairs of TNBC tissues and matched adjacent nontumor tissues. Our results showed that miR-33a levels were much lower in TNBC tissues (Fig. 1b).

### Overexpression of miR-33a inhibits proliferation, mobility and induces G1 cell cycle arrest in TNBC cells

**Fig. 2:**
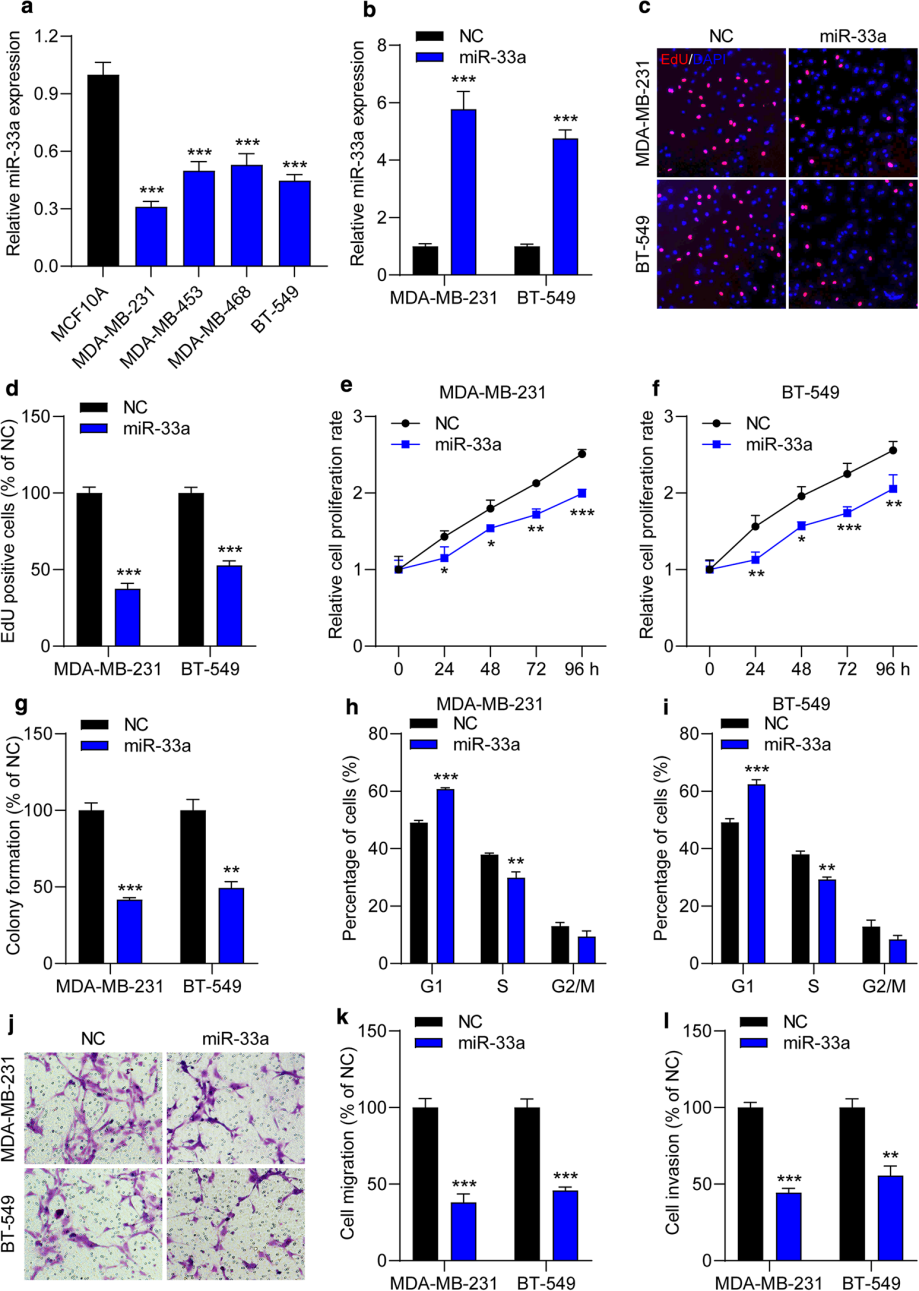
Ectopic expression of miR-33a inhibits the malignant behaviors in TNBC cells. **a** The expression of miR-33a in TNBC cell lines and normal breast epithelial cells was measured by qRT-PCR assay. ***P < 0.001 compared with MCF10A. **b** The expression of miR-33a in TNBC cell lines was elevated after transfection with miR-33a mimics. **c**, **d** MDA-MB-231 and BT-549 cells were transfected with NC or miR-33a mimics for 24 h and cell proliferation was determined by EdU cell proliferation kit. EdU positive cells and images were captured. **e**, **f** The effect of miR-33a on cell proliferation rate was detected by CCK-8 assay. **g** Increased miR-33a reduced colony formation capacities in TNBC. **h**–**l** TNBC cells were transfected with NC or miR-33a mimics for 48 h and then cell cycle analyses were performed by a flow cytometer. **j**, **k** miR-33a mimics decreased the number of migrated **j**, **k** and invasive (**l**) cells. Magnification, ×200. Data are shown as mean ± SEM (n = 3). *P < 0.05, **P < 0.01 and ***P < 0.001 compared with cells transfected with NC mimics groups

Then, to further explore the effect of miR-33a on TNBC cell behaviors, miR-33a mimics were used. We first examined the expression of miR-33a in TNBC cell lines and qRT-PCR assay showed that miR-33a was significantly decreased in 4 TNBC cell lines (MDA-MB-231, MDA-MB-453, MDA-MB-468 and BT-549) than normal MCF10A breast epithelial cells (Fig. 2a). MDA-231 and BT-549 cells were selected for further study due to the much lower miR-33a levels in these cells. We found that the expression of miR-33a increased by 4.5- to 5.5-fold in cells after transfection with miR-33a mimics (Fig. 2b). Then, cell proliferation was determined. We found that ectopic expression of miR-33a significantly decreased the number of EdU-positive cells (Fig. 2c, d). CCK-8 assay also showed that miR-33a suppressed the growth of MDA-MB-231 (Fig. 2e) and BT-549 (Fig. 2f) from 24 to 96 h. In addition, miR-33a overexpression induced a 50% reduction in colony formation capacities of TNBC cells (Fig. 2g). We also found that *CDK4*, a cell proliferation-associated gene, was downregulated in TNBC cells after transfection with miR-33a mimics (Additional file 1: Figure S1A). Then, the effect of miR-33a on cell cycle was detected. Flow cytometric analysis showed that miR-33a mimics induced G1 cell cycle arrest in TNBC cells, but had less effect on the G2/M phase (Fig. 2h, i). Moreover, an in vitro Transwell assay was performed to test the effect of miR-33a on TNBC cells mobility. We found that the migrated and invasion capacities were reduced by nearly 50% in MDA-MB-231 and BT-549 cells transfected with miR-33a mimics (Fig. 2j–l). We also observed *MMP9* mRNA levels were significantly decreased in miR-33a-trasfected cells, compared to cells transfected with NC mimics (Additional file 1: Figure S1B). Overall, miR-33a inhibits proliferation and mobility and induces G1 cell cycle arrest in TNBC cells.

### EZH2 is a direct target of miR-33a

**Fig. 3:**
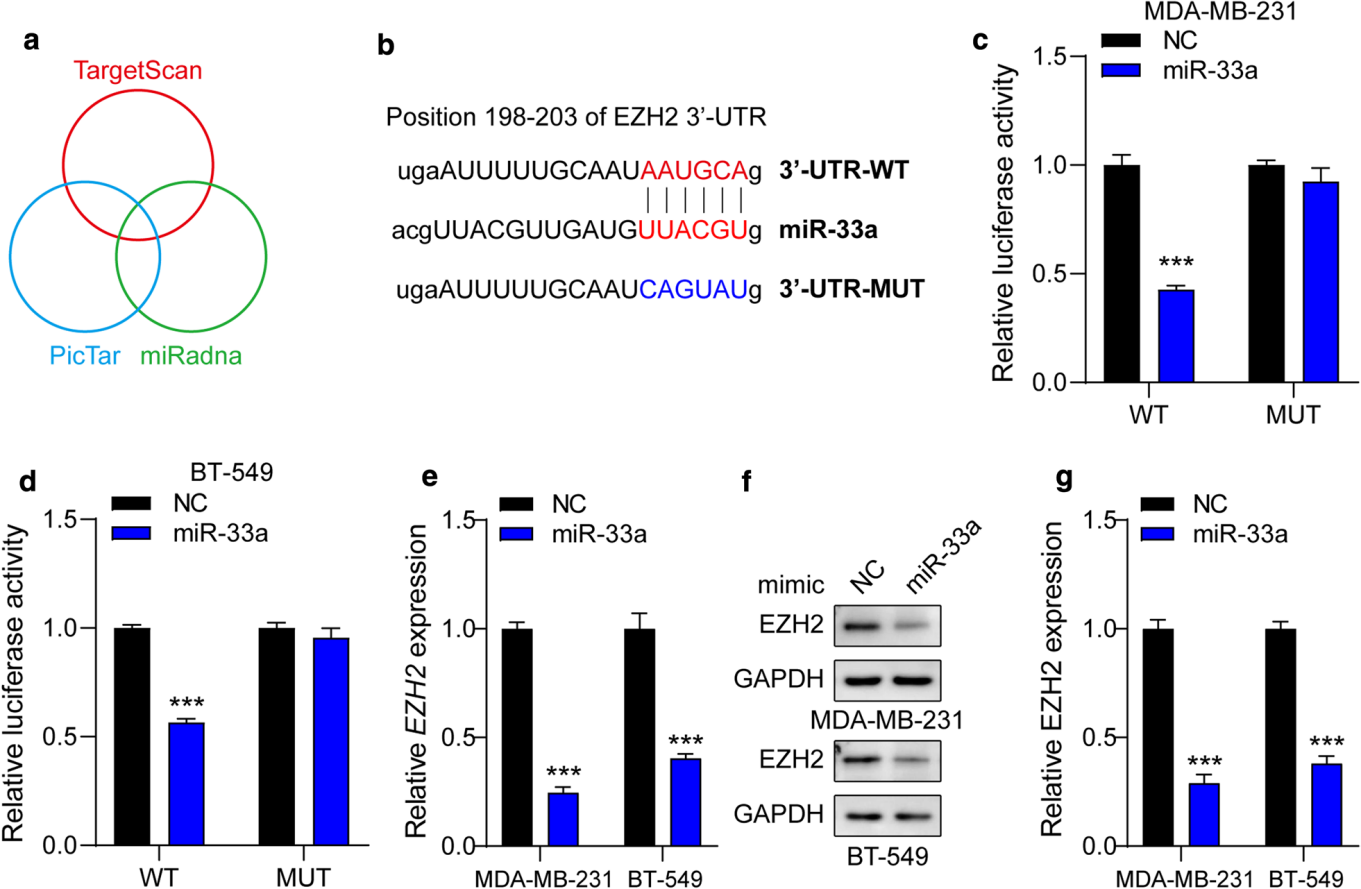
EZH2 is a direct target of miR-33a. **a** The bioinformatic methods used to predict the possible targets of miR-33a. **b** The predicted binding-sites of miR-33a in the 3′-UTR of *EZH2* and its mutated version are shown. **c**, **d** Luciferase reporter assay was performed in MDA-MB-231 (**c**) and BT-549 (**d**) cells co-transfected with the pGL3 construct containing WT or MUT *EZH2* 3′-UTR region and miR-33a mimics. Data were normalized to those from cells co-transfected with the pGL3 and NC mimics. MDA-MB-231 and BT-549 cells were transfected with miR-33a mimics. **e** The EZH2 mRNA expression was detected and normalized to *ACTB*. **f**, **g** Quantitative analysis of Western blots is shown, and GAPDH served as a loading control. Data are shown as mean ± SEM (n = 3). ***P < 0.001 compared with cells transfected with NC mimics groups

Then, we searched for candidate target genes of miR-33a using the databases, TargetScan, miRanda and PicTar (Fig. 3a). We found that a complementary miR-33a sequence was present in the 3′-UTR of *EZH2* mRNA (Fig. 3b), thus, EZH2 was selected for further investigations. Dual luciferase reporter assays showed that miR-33a overexpression clearly inhibited the activity of a luciferase reporter fused to the wild-type (WT) 3′-UTR of *EZH2*, but not of the mutant (MUT) reporter (Fig. 3c, d). In addition, the mRNA and protein levels of EZH2 in MDA-MB-231 and BT-549 cells were also determined. As expected, ectopic expression of miR-33a led to decreases more than 50% in EZH2 mRNA (Fig. 3e) and protein (Fig. 3f, g) expressions. Thus, these results suggest that EZH2 is a direct target of miR-33a in TNBC cells.

### EZH2 is upregulated in TNBC tissues

**Fig. 4:**
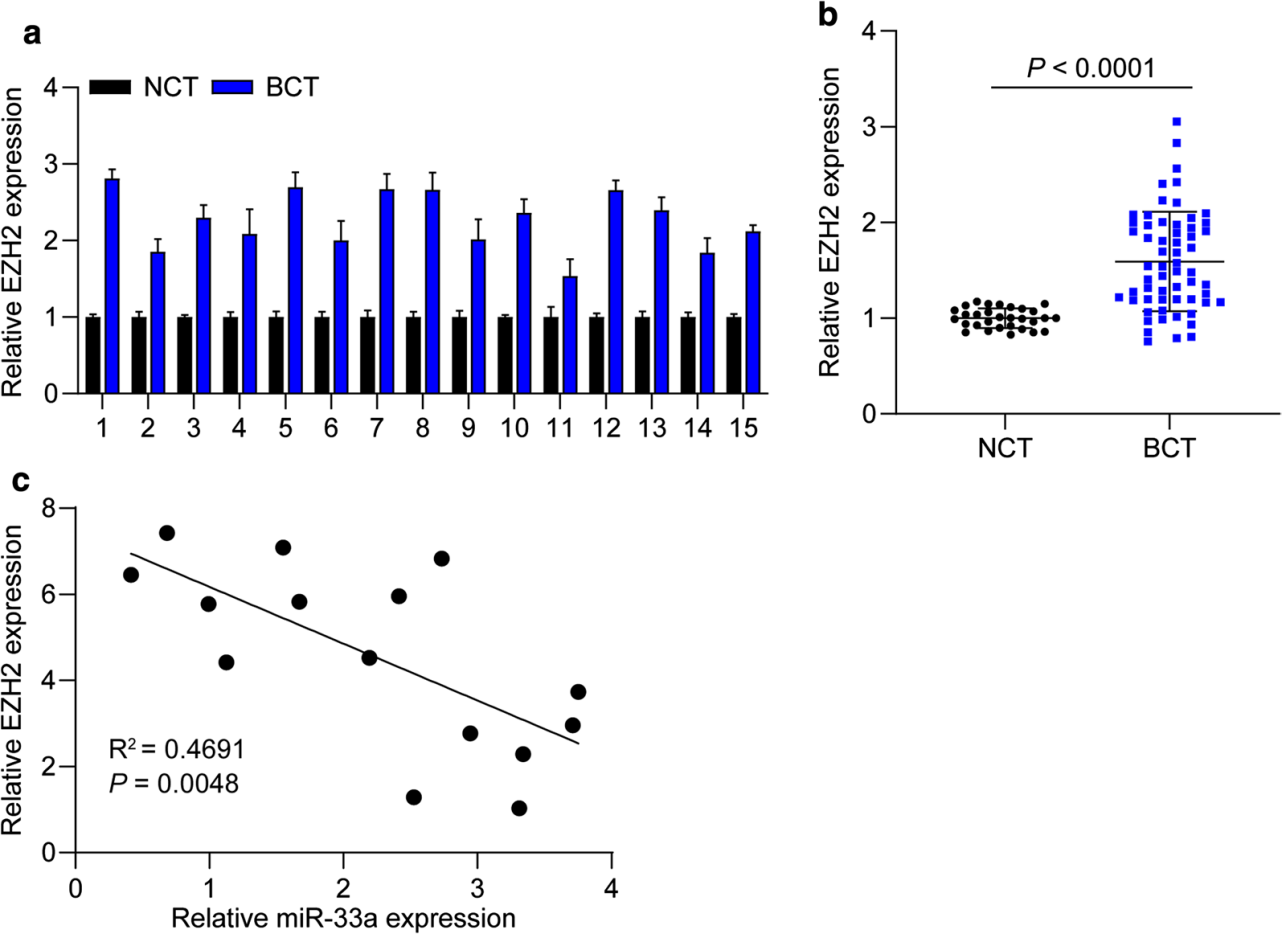
EZH2 is upregulated in TNBC tissues. **a** The expression of EZH2 in 15 TNBC tissues and matched adjacent nontumor tissues was determined using PCR assay. **b** EZH2 levels were significantly higher in TNBC tissues (n = 75) than in adjacent nontumor tissues (n = 30). **c** The correlation between miR-33a and EZH2 expression in TNBC tissues was analyzed. Data are shown as mean ± SEM

To corroborate the correlation between miR-33a and EZH2 in TNBC tissues, we further detected the expression of EZH2 in TNBC tissues and adjacent nontumor tissues. We found that EZH2 expression was remarkably higher in the TNBC tissues than the matched adjacent non-tumor tissues (Fig. 4a. *P* < 0.001). QRT-PCR assay also revealed that the expression EZH2 in TNBC tissues was dramatically higher than adjacent nontumor tissues (Fig. 4b). Furthermore, a significantly negative correlation between miR-33a and EZH2 expression in TNBC tissues was observed (Fig. 4c, R^2^ = 0.4691, *P* = 0.0048). Collectively, the results demonstrate that EZH2 levels are upregulated and reveal a negative correlation with miR-33a expression in TNBC tissues.

### EZH2 knockdown mimics the effects of miR-33a overexpression in TNBC cells

**Fig. 5:**
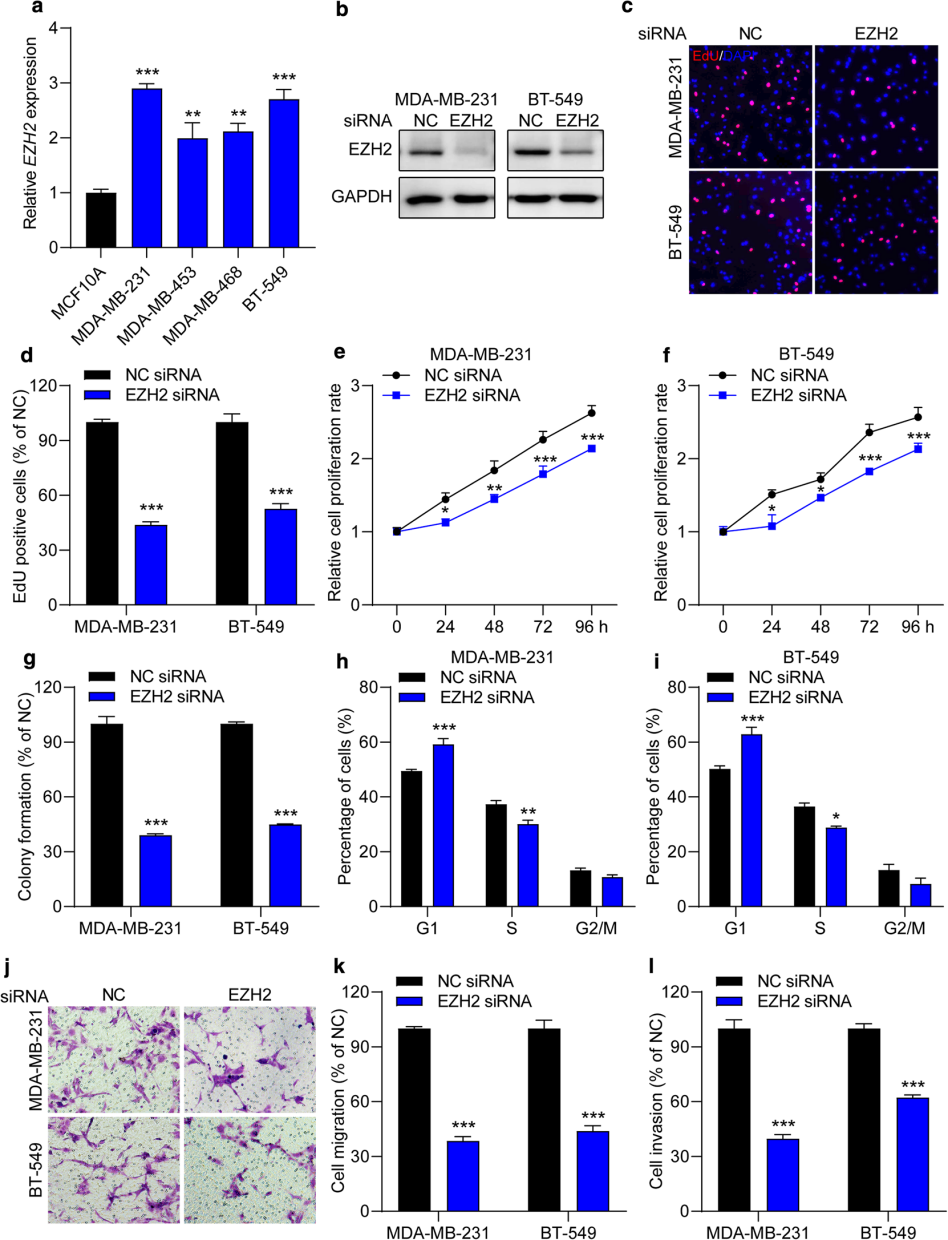
The knockdown of EZH2 suppresses the malignant behaviors in TNBC cells. **a** The expression of EZH2 in TNBC cell lines and normal breast epithelial cell was measured by qRT-PCR assay. **P < 0.01, ***P < 0.001 compared with MCF10A cells. **b** The expression of EZH2 in TNBC cell lines was significantly downregulated after transfection with EZH2 siRNA. **c**, **d** MDA-MB-231 and BT-549 cells were transfected with NC or EZH2 siRNA for 24 h and cell proliferation was determined by an EdU cell proliferation kit. EdU positive cells and images were captured. **e**, **f** The effect of EZH2 on cell proliferation rate was determined by CCK-8 assay. **g** Decreased miR-33a showed attenuated colony formation capacity in TNBC cells. **h**–**l** TNBC cells were transfected with NC or EZH2 siRNA for 48 h and then cell cycle analysis was performed by a flow cytometer. **j**, **k** EZH2 siRNA decreased the number of migrated (**j**, **k**) and invasive (**l**) cells. Magnification, ×200. Data are shown as mean ± SEM (n = 3). *P < 0.05, **P < 0.01 and ***P < 0.001 compared with cells transfected with NC siRNA groups

Next, to verify that whether EZH2 is a key regulator of cellular behaviors in TNBC cells, EZH2 siRNA was used, which mimicked miR-33a overexpression in TNBC cells. We found that EZH2 expression was significantly increased in TNBC cell lines (MDA-MB-231, MDA-MB-453, MDA-MB-468 and BT-549) compared with normal breast epithelial cell line MCF10A (Fig. 5a). Then, MDA-MB-231 and BT-549 cells were transfected with EZH2 siRNA to inhibit EZH2 expression (Fig. 5b, and Additional file 2: Figure S2A). We observed that the number of EdU-positive cells was reduced by 50% to 60% in cells transfected with EZH2 siRNA (Fig. 5c, d). The CCK-8 assay also showed that EZH2 siRNA dramatically inhibited TNBC cell growth from 24 to 96 h (Fig. 5e, f). Moreover, EZH2 inhibition significantly attenuated the colony formation capacity (Fig. 5g) and downregulated *CDK4* mRNA levels (Additional file 2: Figure S2B) in both TNBC cell lines. Flow cytometric analysis revealed that EZH2 downregulation increased the percentage of TNBC cells arrested in G1 phase (Fig. 5h, i). Furthermore. the number of migrated (Fig. 5j, k) and invasive (Fig. 5l) cells was decreased 40% to 65% after transfection with EZH2 siRNA compared with NC siRNA. Moreover, the knockdown of EZH2 also downregulated the mRNA and protein levels of MMP9 expression in MDA-MB-231 and BT-549 cells (Additional file 2: Figure S2C). Taken together, EZH2 inhibition suppresses proliferation and mobility and induces G1 cell cycle arrest in TNBC cells.

### EZH2 is the key mediator of the effects of miR-33a in TNBC cells

**Fig. 6:**
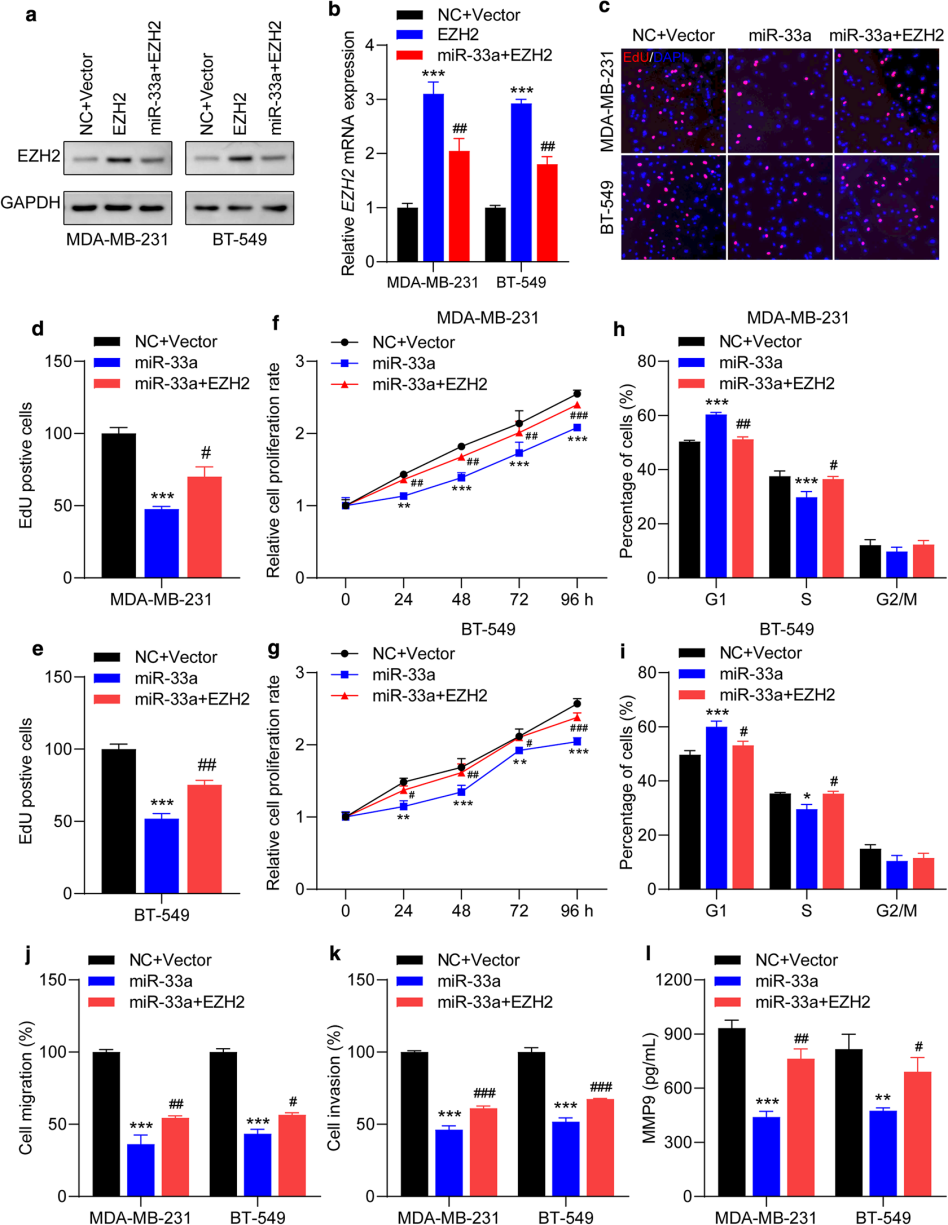
miR-33a-mediated suppressive effects on malignant behaviors in TNBC cells can be offset by EZH2 overexpression. **a**, **b** The expression of EZH2 in TNBC cells after indicated transfected was determined by Western blot and qRT-PCR assays. **a** Representative blots and **b** the levels of *EZH2* mRNA after in cells indicated transfections are shown. **c**–**e** Ectopic expression of EZH2 counteracted miR-33a-mediated reduction of EdU incorporation in TNBC cells. Representative images (**c**) and the number of EdU-positive MDA-MB-231 (**d**) and BT-549 (**e**) cells are shown. **f**, **g** EZH2 overexpression promoted the proliferation rate in MDA-MB-231 and BT-549. **h**, **i** Increased EZH2 reduced the percentage of cells arrested in G1 cell cycle mediated by miR-33a. **j**, **k** MiR-33a-induced decreases in cell migration and invasion capacities can be enhanced by ectopic expression of EZH2. **l** EZH2 overexpression promoted the secret of MMP9 in TNBC cells. Data are shown as mean ± SEM (n = 3). *P < 0.05, **P < 0.01 and ***P < 0.001 compared with cells transfected with NC + Vector groups; ^#^*P* < 0.05, ^##^*P* < 0.01 and ^###^*P* < 0.001 compared with cells transfected with miR-33a mimic groups

To further confirm that EZH2 is a direct target of miR-33a, MDA-MB-231 and BT-549 were co-transfected with EZH2 overexpressing plasmid and miR-33a mimics. We first detected that transfection efficiency of the EZH2 overexpression plasmid in TNBC cells. As shown in Fig. 6a, b, the expression levels of EZH2 protein and mRNA were significantly upregulated in MDA-MB-231 and BT-549 cells after transfection with EZH2 overexpression plasmid, while can be attenuated by miR-33a mimics. Then, cell proliferation was first examined. We found that the number of EdU-positive cells in co-transfection groups was dramatically higher than cells transfected with miR-33a mimics (Fig. 6c–e). Compared to cells after transfection with miR-33a mimics, the proliferation rate is dramatically elevated in cells co-transfected with EZH2 overexpressing plasmid and miR-33a mimics (Fig. 6f, g). EZH2 overexpression also reversed the *CDK4* mRNA decrease induced by miR-33a mimics (Additional file 3: Figure S3A). Furthermore, ectopic expression of EZH2 significantly decreased the number of TNBC cells arrested in G1 cell cycle (Fig. 6h, i). The number of migrated and invasive cells, and the expression levels of MMP9 mRNA and protein were significantly decreased in cells transfected with miR-33a mimics, which can be reversed by ectopic expression of EZH2 (Fig. 6j–l, and Additional file 3: Figure S3B). Together, these results demonstrate that EZH2 overexpression antagonizes miR-33a-mediated inhibitory effect on TNBC cell behaviors, which implies that EZH2 is a downstream target of miR-33a.

## Discussion

Increased evidence supports that noncoding RNAs, including miRNAs, lncRNAs and circRNAs, are important regulators in tumor development, growth, metastasis and angiogenesis [31, 32]. MiRNAs can be oncogenic miRNAs and tumor-suppressive miRNAs [33, 34]. It was reported that miR-33a was downregulated in a variety of cancers, and ectopic expression of miR-33a suppressed multiple malignant behaviors [15–18]. miR-33a can suppress epithelial-mesenchymal transition and metastasis in non-small cell lung cancer, and its expression in the blood of lung cancer patients was lower than that in healthy controls; thus, it could be regarded as a tumor suppressor and a novel biomarker for the diagnosis of lung cancer [15, 35]. It also targets ST8SIA1 to suppress colorectal cancer progression [36] and targets HIF-1α to inhibit melanoma cell growth and mobility [37]. Regarding breast cancer, decreased miR-33a in breast cancer tissues was associated with lymph node metastasis, and the expression of miR-33a was dramatically lower in metastatic breast cancer cell lines than in nonmetastatic cancer cell lines and normal breast epithelial cells [18]. Further study showed that miR-33a overexpression inhibited metastatic breast cancer cell growth and mobility *in vitro* and *in vivo* by targeting ADAM9 and ROS1 [18]. Therefore, miR-33a acts as a tumor-suppressive miRNA in most kinds of cancer. However, Wang H reported that upregulated expression of miR-33a was correlated with poor prognosis of GBM patients and enhanced self-renewal of glioma-initiating cells via activation of the PKA and NOTCH pathways by targeting PDE8A and UVRAG [38]. Thus, miR-33a can act as either oncogenic or tumor-suppressive miRNA, which may depend on the tumor type. Nevertheless, the expression profiles and functions of miR-33a in triple-negative breast cancer growth and metastasis are less clear. In our study, we found that miR-33a was significantly downregulated in TNBC tissues compared with adjacent nontumor tissues, and further studies revealed that ectopic expression of miR-33a inhibited TNBC cell proliferation, colony formation, migration and invasion and induced G1 cell cycle arrest. miR-33a overexpression also downregulated TNBC cell *CDK4* and *MMP9* mRNA levels, which may account for the suppression of proliferation and mobility in TNBC cells.

EZH2, as a methyltransferase and component of PRC2, regulates H3K27 methylation to mediate gene transcriptional silencing [20]. EZH2 was higher in tumor tissues than in adjacent nontumor tissues and was associated with poor prognosis in tumor patients [39–41]. In breast cancer, its expression was correlated with breast cancer aggressiveness and could serve as an independent predictor of survival and recurrence [42]. A recent study reported that EZH2 promoted the mobility of TNBC cells by regulating the TIMP-2/MMP-2/9 pathway [23]. miRNAs were reported to regulate EZH2 expression in TNBC. Wu QJ reported that miR-1301 inhibited TNBC cell proliferation, migration and colony formation as well as xenograft growth by negatively regulating EZH2 expression [28]. Another study revealed that EZH2 reduction mediated by miR-340 mimic induced decreased expression of DNM1, H3K27me3, β-catenin and p-STAT3, which led to inhibition of miR-21 activity and upregulation of miR-200a/b, which contributed to inhibition of TNBC progression [25]. Here, our results showed that EZH2 expression was negatively correlated with miR-33a in TNBC tissues and was significantly higher in TNBC cell lines. Moreover, a luciferase reporter assay corroborated that miR-33a directly targeted EZH2 and inhibited EZH2 expression. EZH2 inhibition by EZH2 siRNA exerted similar effects on TNBC cell behaviors, while EZH2 upregulation by transfection with an EZH2-overexpressing plasmid reversed the suppressive effects mediated by miR-33a.

Many factors are responsible for the downregulation of tumor-suppressive miRNAs [43, 44]. It was reported that miR-33a downregulation in tumors may be associated with upregulation of proto-oncogenic lncRNAs, and this study showed that miR-33a inhibition mediated by lncRNA DANCR promoted osteosarcoma development and cancer stemness characteristics by inducing Axl upregulation [45]. EZH2 was also reported to induce silencing of tumor-suppressive miRNA I tumor cells, and miR-34a was epigenetically silenced by EZH2, which promoted cholangiocarcinoma cell growth by activating the Notch pathway [46]. Therefore, whether miR-33a downregulation in TNBC is mediated by EZH2 or oncogenic lncRNA remains to be further investigated.

Given that EZH2 was overexpressed in many types of cancer, and inhibitors targeting EZH2 were also developed. Tazemetostat, an EZH2 inhibitor, has been approved for treating epithelioid sarcoma, and it is the first EZH2 inhibitor approved by FDA [47]. Other EZH2 inhibitor, GSK343 and GSK236, were also reported to inhibit tumor progression in various cancer, such as glioblastoma [48], head and neck cancer, [49] and breast cancer [50]. Our in vitro study confirmed the regulatory mechanism of miR-33a/EZH2 cascade in TNBC progression, thus, more investigation of the effects of miR-33a and EZH2 on tumor growth and metastasis *in vivo* is needed. Moreover, EZH2 inhibitors can be used for in vitro and in vivo studies, which may further provide a rationale for potential therapeutic strategy for the treatment of TNBC patients.

## Conclusions

In the current study, we verified an important role of miRNA-33a/EZH2 cascade in TNBC progression. We found that the expression of miR-33a was downregulated in TNBC tissues and cell lines, and it also had an inverse correlation with EZH2 expression in TNBC tissues. Our study confirmed that miR-33a was a tumor-suppressive miRNA in TNBC and indicated that EZH2 can be a potential therapeutic target for TNBC treatment.

## Supplementary information

**Additional file 1: Fig.** **1** MiR-33a overexpression inhibited the levels of *CDK4* and *MMP9* mRNA expression in TNBC cells. TNBC cell were transfected with miR-33a mimics for 24 h, and *CDK4* (A) and *MMP9* (B) mRNA levels were determined by qRT-PCR assay. Data are shown as mean ± SEM (n = 3). *P < 0.05, **P < 0.01 and ***P < 0.001 compared with cells transfected with NC mimics groups.

**Additional file 2: Fig.** **2** EZH2 siRNA downregulated the expression levels of EZH2, CDK4 and MMP9 in TNBC cells. (A) TNBC cells were transfected with different sets EZH2 siRNAs for 24 h and transfection efficiency was determined by qRT-PCR assay. (B-C) EZH2 siRNA downregulated the levels *CDK4* mRNA (B), and *MMP9* mRNA and protein (B) in TNBC cells. Data are shown as mean ± SEM (n = 3). ***P < 0.001 compared with cells transfected with NC mimics groups.

**Additional file 3: Fig.** **3** Ectopic expression of EZH2 attenuated miR-33a-induced downregulation of CDK4 and MMP9 expression. (A-B) TNBC cells were co-transfected with miR-33a and EZH2 overexpressing plasmid for 24 h, and the expression of *CDK4* (A) and *MMP9* (B) mRNA were measured by qRT-PCR assay. Data are shown as mean ± SEM (n = 3). ***P < 0.001 compared with cells transfected with NC+Vector groups; ^##^*P* < 0.01 and ^###^*P* < 0.001 compared with cells transfected with miR-33a mimics groups.

## Data Availability

The analyzed data sets generated during the study are available from the corresponding author on reasonable request.

## Abbreviations

3′-UTR: 3′-Untranslated regions
BC: Breast cancer
CCK8: Cell counting kit-8
EdU: 5-Ethynyl-2-deoxyuridine
ER: Estrogen receptor
EZH2: Enhancer of zeste homolog-2
HER-2: Human epidermal growth factor receptor 2
PI: Propidium iodide
PR: Progesterone receptor
PVDF: Polyvinylidene difluoride
SDS-PAGE: Polyacrylamide gel electrophoresis
TNBC: Triple negative breast cancer
