# Supplementary figures and images for: MiR-33a functions as a tumor suppressor in triple-negative breast cancer by targeting EZH2
Source: Cancer Cell Int. 2020 Mar 18;20:85. doi: 10.1186/s12935-020-1160-z (PMC7079399; doi:10.1186/s12935-020-1160-z)

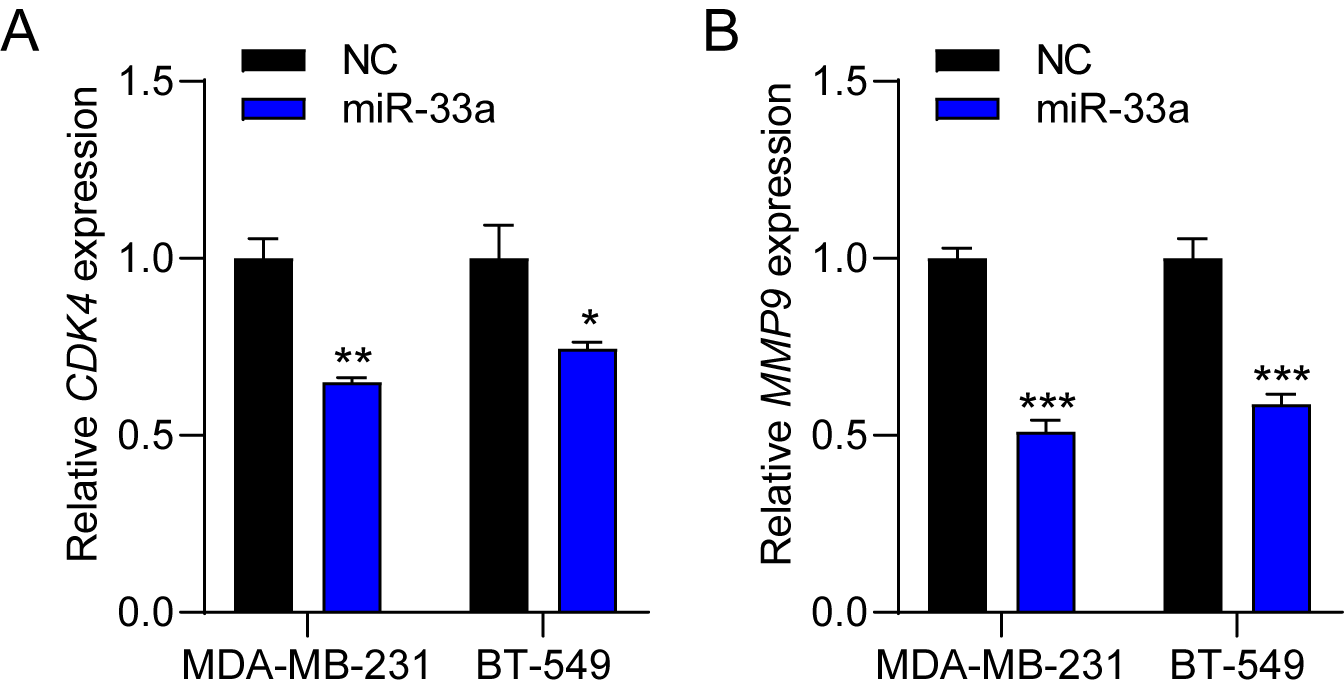

Supplement: Supplementary file 1 — Additional file 1: Fig. 1 MiR-33a overexpression inhibited the levels of CDK4 and MMP9 mRNA expression in TNBC cells. TNBC cell were transfected with miR-33a mimics for 24 h, and CDK4 (A) and MMP9 (B) mRNA levels were determined by qRT-PCR assay. Data are shown as mean ± SEM (n = 3). *P < 0.05, **P < 0.01 and ***P < 0.001 compared with cells transfected with NC mimics groups. [file 12935_2020_1160_MOESM1_ESM.tif]

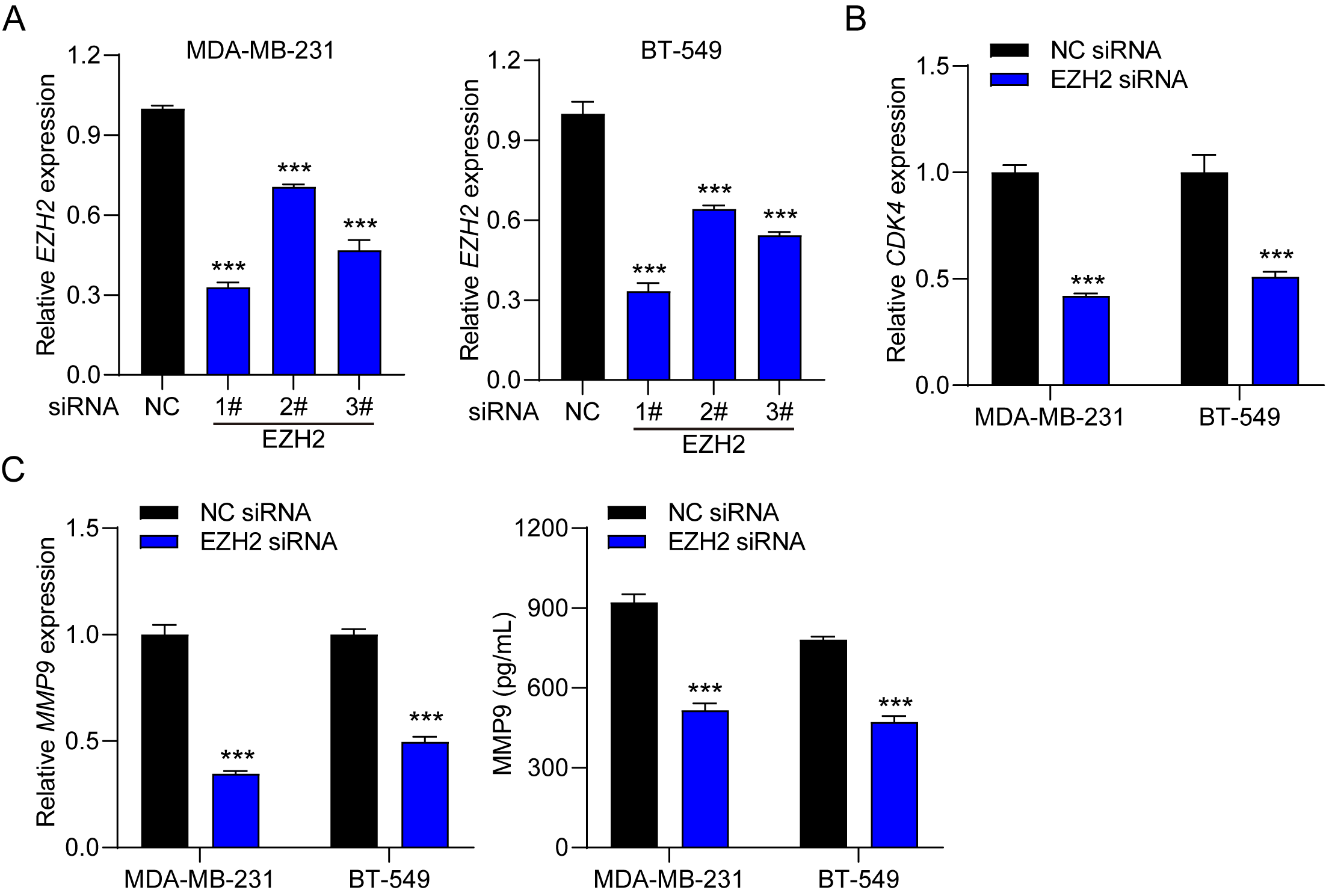

Supplement: Supplementary file 2 — Additional file 2: Fig. 2 EZH2 siRNA downregulated the expression levels of EZH2, CDK4 and MMP9 in TNBC cells. (A) TNBC cells were transfected with different sets EZH2 siRNAs for 24 h and transfection efficiency was determined by qRT-PCR assay. (B-C) EZH2 siRNA downregulated the levels CDK4 mRNA (B), and MMP9 mRNA and protein (B) in TNBC cells. Data are shown as mean ± SEM (n = 3). ***P < 0.001 compared with cells transfected with NC mimics groups. [file 12935_2020_1160_MOESM2_ESM.tif]

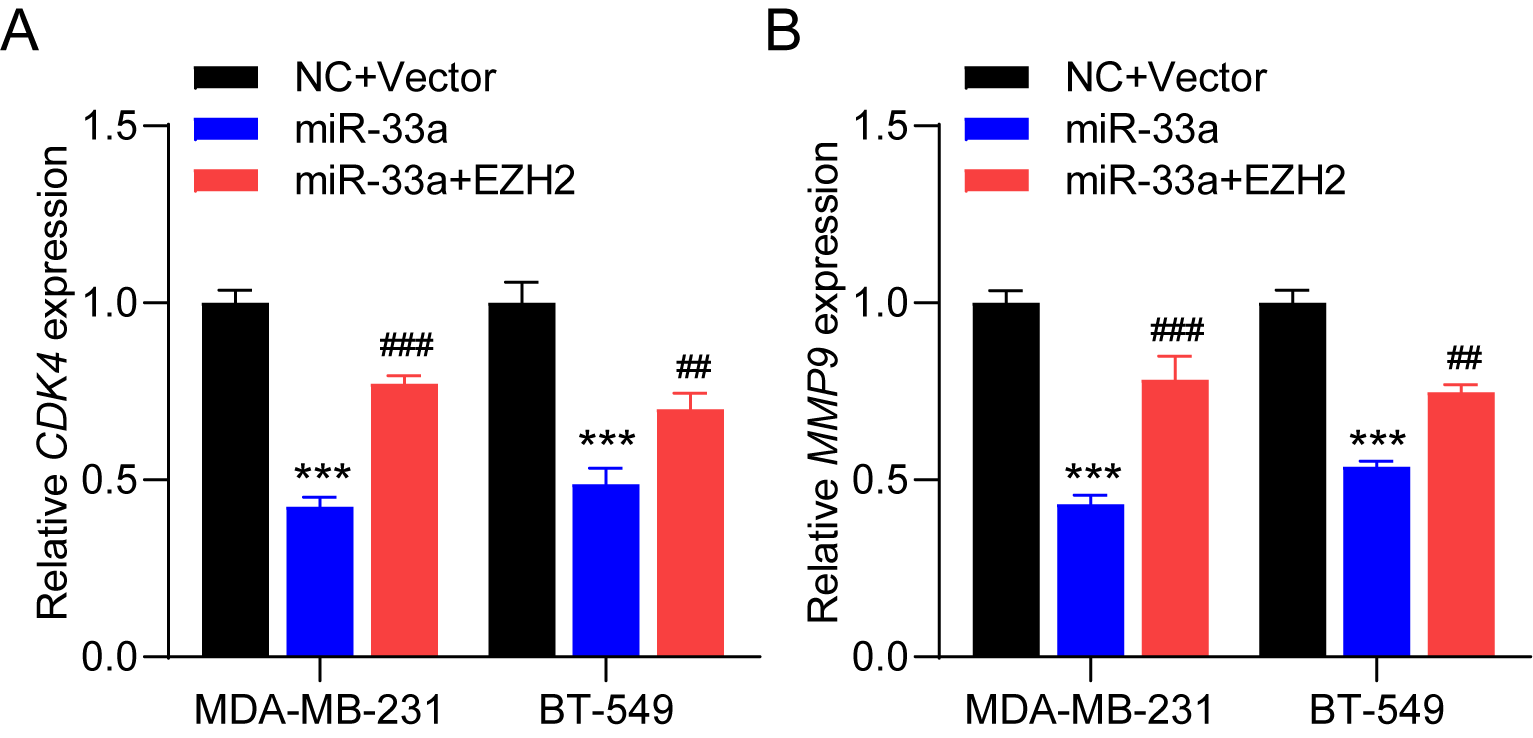

Supplement: Supplementary file 3 — Additional file 3: Fig. 3 Ectopic expression of EZH2 attenuated miR-33a-induced downregulation of CDK4 and MMP9 expression. (A-B) TNBC cells were co-transfected with miR-33a and EZH2 overexpressing plasmid for 24 h, and the expression of CDK4 (A) and MMP9 (B) mRNA were measured by qRT-PCR assay. Data are shown as mean ± SEM (n = 3). ***P < 0.001 compared with cells transfected with NC+Vector groups; ##P < 0.01 and ###P < 0.001 compared with cells transfected with miR-33a mimics groups. [file 12935_2020_1160_MOESM3_ESM.tif]
